# Analysis of *MLH3* C2531T polymorphism in Iranian women with unexplained infertility 

**Published:** 2013-01

**Authors:** Hossein Pashaiefar, Mohammad Hasan Sheikhha, Seyyed Mehdi Kalantar, Tahereh Jahaninejad, Mohammad Ali Zaimy, Nasrin Ghasemi

**Affiliations:** 1 *Department of Medical Genetics, Medical Faculty, Shahid Sadoughi University of Medical Sciences, Yazd, Iran.*; 2 *Department of Medical Genetics, Research and Clinical Center for Infertility, Shahid Sadoughi University of Medical Sciences, Yazd, Iran.*

**Keywords:** *Female infertility*, *Mismatch repair genes*, *MLH3*, *Polymorphism*

## Abstract

**Background: **Meiotic genes are very important candidates for genes contributing to female and male infertility. Mammalian MutL homologues have dual roles in DNA mismatch repair (MMR) after replication errors and meiotic reciprocal recombination. The MutL homologs, *MLH1* and *MLH3*, are crucial for meiotic reciprocal recombination and human fertility. In this study the functional polymorphisms of *MLH3* C2531T was investigated in Iranian women with unexplained infertility.

**Objective:** Investigating the association between a common SNP (single nucleotide polymorphism) C2531T in the *MLH3* gene and female infertility.

**Materials and Methods: **In total, 105 women with unexplained infertility as case group and 100 women with at least one child and no history of infertility or abortion as controls were recruited for this association study. The *MLH3* C2531T polymorphism was tested by tetra-amplification refractory mutation system-PCR (4P-ARMS-PCR) method.

**Results:** The *MLH3* 2531C and T alleles frequencies were 43.33% and 56.67% among infertile patients, and 61.5% and 38.5% among normal controls, respectively. In the patient and control subjects the CC (Pro 844 Pro) genotype frequency of *MLH3* C2531T was 4.76% and 25%, the CT (Pro 844 Leu) genotype was 77.15% and 73%, and the TT (Leu 844 Leu) genotype was 19% and 2%, respectively (p=0.0001).

**Conclusion:** The presence of the polymorphic allele T leads to an increased risk of 2.09 times (OR=2.09, 95% CI=1.38-3.16; p=0.0001) for developing infertility in relation to the control group. Therefore, our data suggest that the *MLH3* C2531T polymorphism can be associated with the risk of unexplained infertility in Iranian women.

## Introduction

Infertility is described as the inability of a sexually active couple, not using any birth control to get pregnant after one year of trying. It is estimated that about 15% of couples in the world suffer from infertility (1). 

The etiology of many cases of infertility remains poorly understood. It is obvious that infertility causes are heterogeneous because a number of factors contribute to reproductive success. About 30% of couples with reproductive problem are diagnosed with unexplained infertility (2). Current evidence suggests that genetic factors contribute to the etiology of female infertility in humans (3). 

Meiosis is the fundamental feature of sexually reproducing organisms. It is the cell division that produces haploid gametes from diploid cells. Its molecular regulation has been conserved throughout eukaryotic evolution. Meiotic genes are very important candidates for genes contributing to female infertility (4). The most important stage of meiosis is prophase I, in which homologous chromosomes pair and remains tethered until the first meiotic division, when they must segregate equally into daughter cells that then enter meiosis II. 

Mismatch repair (MMR) contributes to maintenance of genomic integrity and for correcting DNA mismatch after DNA replication in somatic cells while it participates in crossing over between homologous chromosomes in meiosis. A meiosis-specific mismatch protein, MSH4, heterodimerises with MSH5 appears at recombination sites at zygonema (5-7). At mid-pachynema, MSH4 interacts with MLH1 and MLH3 (8). The MLH1 and MLH3 proteins are associated with late recombination nodules that are known to correlate with crossover sites (8, 9). 

The understanding of the mechanisms of meiosis and fertility has mainly benefitted from knockout and transgenic mouse models that exclusively have reproduction failure. Both male and female Mlh1-/- and Mlh3-/- mice are infertile. Males of both genotypes arrest in the metaphase I. Mlh1-/- oocytes fail to complete the meiosis II after fertilization with normal sperms, whereas a few numbers of abnormal Mlh3-/- oocytes extrude both the first and second polar body (10-13). 

The human gene of *MLH3* was first identified in 2000. The human *MLH3* gene is located on the long arm of the chromosome 14 (14q24.3) with a coding length of 4.3 kb, and has 12 exons. Exon 1 of this gene is 3.3 kb, accounting for 75% of the coding region (14). A total of 22 variants have been identified in* MLH3* gene. Overall 12 of the 22 variants were missense changes, including K231Q, F390I, P551S, V420I, R647C, Y720C, R797H, N826D, E828D, H823Y, P844L, and T942I (15). 

The majority of the missense variants occurred in a region of *MLH3* that has no homology with other DNA mismatch repair genes. Four variants of R647C, E828D, P844L and T942I in *MLH3* gene are predicted to affect function of protein by in silico analysis using the SIFT algorithm (16). *MLH3* protein plays a central role in meiotic recombination and it is reasonable to hypothesize that mutations in the *MLH3* gene may be associated with male and female infertility.

Although there are no studies about mutations of the *MLH3* gene in the female infertile cases, these mutations are likely to be causative for female infertility, because infertility phenotype is observed previously in female mice with an Mlh3 deficiency (11). The present study, evaluated, for the first time, the relationship of the C2531T polymorphism of the *MLH3* gene with female infertility in a case-control study.

To investigate this relationship, we studied the frequency of distribution of the common SNP C2531T in the *MLH3* gene in 105 infertile patients and compared the results with 100 fertile controls in order to explore the possible association between gene variation and female infertility.

## Materials and methods

**Subjects**

A total of 105 Iranian women with unexplained infertility were enrolled in this study. Infertile patients of known cause, such as hormonal, structural, immunological, and coagulation abnormalities, were excluded. All of the patients were examined for detection of anatomic abnormalities of the genital tract. Thrombophilia screening was performed with the measurement of plasma levels of antithrombin and proteins C and S. Blood tests for hyperthyroidism, diabetes mellitus, and hyperprolactinemia were also performed. 

All patients and their partners have a normal karyotype. Blood samples were drawn at the clinic after appropriate informed consent was obtained. A total of 100 blood samples from Iranian women with at least one child and no history of infertility or miscarriage were used as controls. The study was approved by the Yazd Research and Clinical Centre for Infertility Ethical Committee, and informed consent was taken from each patient. This work was supported by Yazd Research and Clinical Center for Infertility.

**DNA extraction and genotyping of** ***MLH3*** **C2531T polymorphism**

Genomic DNA was isolated from peripheral blood samples using the standard salting-out procedure. *MLH3* C2531T polymorphism was analyzed using the tetra-amplification refractory mutation system-PCR (4P-ARMS-PCR) method (12). The primers used in the 4P-ARMS-PCR of DNA fragments included one pair of outer primers and one pair of inner primers are showed in table I (13). The PCR reaction was carried out in a final volume of 25 μL, containing 1X buffer, 2.5 mM of MgCl_2_, 5 pmol of primers *MLH3* F1 and *MLH3* R1, 20 pmol of primers *MLH3* F2 and *MLH3* R2, 0.1 mM of each dNTP, 1U Taq Polymerase (Invitrogen), and 200 ng of DNA.

**Figure 1:**
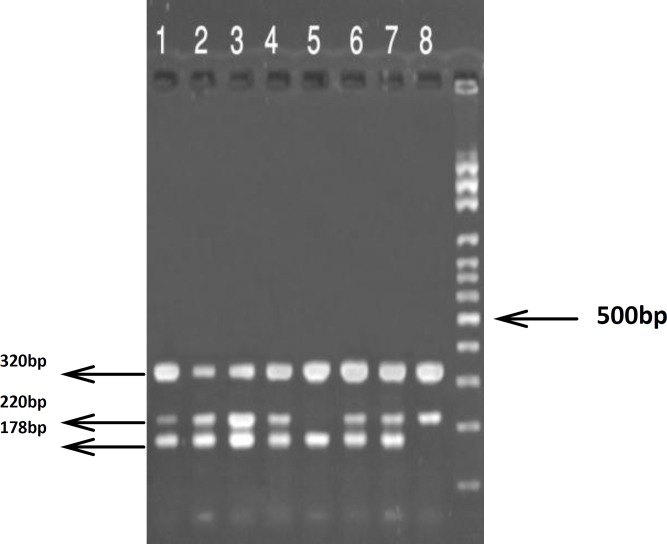
The (4P-ARMS-PCR) analysis of *MLH3* C2531T polymorphism.

The touch-down amplification was performed with an initial melting step of 94^o^C for 5 min; followed by 20 cycles of 94^o^C for 30 s, annealing temperature starting at 65^o^C for 30 s (decreasing 0.5^o^C per cycle), and 72^o^C for 30s for extension. This step was followed by 10 cycles of 94^o^C for 30 s, 55^o^C for 30 s, 72^o^C for 30 s, and a final elongation step of 5 min at 72^o^C. The PCR product was visualized on 3.0% agarose gel containing ethidium bromide under UV light. The C allele generated a 178 bp band, and the T allele generated a 220 bp band, although they had a common 320 bp band amplified by outer primers (*MLH3* F1 and *MLH3* R1) (Figure 1).

**Statistical analysis**

The allele and genotype frequency of the infertile patients and normal controls were calculated by counting. The chi-square test was used to compare allele and genotype frequencies between patient and normal groups and to estimate the Hardy-Weinberg equilibrium. Statistical tests of significance and χ2 analysis were performed using SPSS statistic software version16.0.The differences in allelic and genotypic frequencies of C2531T locus in the *MLH3* gene between case and control groups were evaluated by χ2 test with odds ratio (OR).

## Results

The genotype and allele frequencies of *MLH3* C2531T polymorphism are showed in Table II. The *MLH3* 2531C and T alleles frequencies were 43.33% and 56.67% among infertile patients, and 61.5% and 38.5% among normal controls. Tests for Hardy-Weinberg equilibrium for the studied SNP was performed, and the null hypothesis of Hardy-Weinberg equilibrium was not rejected (p=0.0001). The genotype frequency of *MLH3* C2531T polymorphisms in our study subjects were significantly different between the patients with unexplained infertility and normal controls (p=0.0001). In specific, in the patient and control subjects the CC (Pro 844 Pro) genotype frequency of *MLH3* C2531T was 4.76% and25%, the CT (Pro 844 Leu) genotype was 77.15% and 73% and the TT (Leu 844 Leu) genotype was 19% and 2%, respectively. In the present study, there was a statistically significant difference between the groups of infertile women compared to the control group (p=0.0001) with regard to the *MLH3* C2531T polymorphism, suggesting that this polymorphism might be related to women infertility. The presence of the polymorphic allele T leads to an increased risk of 2.09 times (OR=2.09, 95% CI=1.38-3.16) to develop infertility in relation to the control group.

**Table I:** The primers used in the 4P-ARMS-PCR

**Primers**	**Sequence**
*MLH3* F1	[5′ACCAATCTCAGTCTTCAAGTTGAACCTG3′]
*MLH3 *R1	[5′ATCATCCCCATTGTTTGAGTTTCTCTTT3′]
*MLH3* F2	[5′GGATGAAGATTGTTTAGAACAACAGATTCC 3′]
*MLH3* R2	[5′GGGTCATAGGACTTTCTCTCAAACGAA 3′]

**Table II:** Genotype and allele frequencies of the *MLH3* C2531T (Pro844Leu) polymorphism in female with unexplained infertility and fertile controls

**Genotypes and alleles**	**Infertile patients (n=105) ** **n (%)**	**Controls (n=100) ** **n (%)**	**OR (95% CI)**
CC	5 (4.76)	25 (25)	
CT	81 (77.15)	73 (73)	
TT	19 (18.1)	2 (2)	
C	91 (43.33)	123 (61.5)	
T	119 (56.67)	77 (38.5)	2.09 (1.38-3.16)

## Discussion

Our results regarding analyzes of 105 patients and 100 controls, showed that the *MLH3* C2531T polymorphism was associated with increased risk for the female infertility. In other studies it was shown that both male and female of Mlh1-/- and Mlh3-/- mice are sterile. Males of both genotypes arrest in the metaphase of meiosis I. While Mlh1-/- oocytes fail to complete the meiosis II, whereas a small proportion of abnormal Mlh3-/- oocytes extrude both the first and second polar body (10, 11). 

Interestingly, men with missense mutation (C2531T) in *MLH3* gene have an increased risk of infertility (13). Altogether, these data underline the importance of *MLH3* for human fertility. Recent researches demonstrated that screenings of *MLH3* in testicular tissue from 13 patients with spermatogenic arrest revealed 4 missense and 8 intronic *MLH3* variants, including 2896T/C and 2531C/T. 2896T/C was found in 1 out of the 13 patients, 2531C/T was found in 3 out of 13 cases (17). Therefore, the *MLH3* protein has a crucial role in human fertility.

In the present study, we found an association between the SNP C2531T (P844L) in the *MLH3* gene and female unexplained infertility in blood samples, suggesting that this mutation may be an important genetic risk factor for female infertility in Iran. The MMR (Mismatch repair) system plays a crucial role in all organisms because it maintains the genome integrity during repeated duplication. 

MMR system is composed of several highly-conserved proteins whose functions in repair of mismatched DNA after replication have been demonstrated. Multiple functions, in addition to mismatch repair during replication, have been identified for MMR proteins, such as promotion of meiotic crossover between homologous chromosomes (18-20). 

Meiotic homologous recombination begins when the double-strand breaks (DSBs) are formed in DNA by Spo11 protein. Holliday junction (formed during homologous recombination) resolvase is activated by MSH4-MSH5 and MLH1-MLH3 in the same pathway (21). Mammalian meiosis is different from meiosis in lower eukaryotes. The mechanism of participation of MMR proteins in mammalian meiotic recombination is unknown. 

Our findings in the present study are biologically plausible, based on the previously reported function of MMR components. Cytological studies in mice have shown that *MLH3* is associated with recombination nodules at early pachytene. This study demonstrated that there was an association between a functional polymorphism and female infertility. This finding can be an indication of this fact that the MLH1–*MLH3* pathway plays an important role in making crossovers during mammalian meiosis and female fertility. 

Therefore, the *MLH3* gene polymorphism may be a genetic risk factor for unexplained infertility in women. The further functional study will continue to provide new evidence of the role of the *MLH3* in pathogenesis of female infertility and will help to further elucidate the mechanisms of meiotic recombination in mammal. 

## References

[1] Randolph JF Jr (2000). Unexplained infertility. Clin Obstet Gynecol.

[2] Quaas A, Dokras A (2008). Diagnosis and treatment of unexplained infertility. Rev Obstet Gynecol.

[3] Matzuk MM, Lamb DJ (2008). The biology of infertility: research advances and clinical challenges. Nat Med.

[4] Sanderson ML, Hassold TJ, Carrell DT (2008). Proteins involved in meiotic recombination: a role in male infertility?. Sys Biol Reprod Med.

[5] Edelmann W, Cohen PE, Kneitz B, Winand N, Lia M, Heyer J (1999). Mammalian MutS homologue 5 is required for chromosome pairing in meiosis. Nat Genet.

[6] Bocker T, Barusevicius A, Snowden T, Rasio D, Guerrette S, Robbins D (1999). hMSH5: a human MutS homologue that forms a novel heterodimer with hMSH4 and is expressed during spermatogenesis. Cancer Res.

[7] Santucci-Darmanin S, Walpita D, Lespinasse F, Desnuelle C, Ashley T, Paquis-Flucklinger V (2000). MSH4 acts in conjunction with MLH1 during mammalian meiosis. FASEB J.

[8] Marcon E, Moens P (2003). MLH1p and MLH3p Localize to Precociously Induced Chiasmata of Okadaic-Acid-Treated Mouse Spermatocytes. Genetics.

[9] Moens PB, Kolas NK, Tarsounas M, Marcon E, Cohen PE, Spyropoulos B (2002). The time course and chromosomal localization of recombination-related proteins at meiosis in the mouse are compatible with models that can resolve the early DNA-DNA interactions without reciprocal recombination. J Cell Sci.

[10] Edelmann W, Cohen PE, Kane M, Lau K, Morrow B, Bennett S (1996). Meiotic pachytene arrest in MLH1-deficient mice. Cell.

[11] Lipkin SM, Moens PB, Wang V, Lenzi M, Shanmugarajah D, Gilgeous A (2002). Meiotic arrest and aneuploidy in MLH3-deficient mice. Nat Genet.

[12] Ye S, Dhillon S, Ke X, Collins AR, Day IN (2001). An efficient procedure for genotyping single nucleotide polymorphisms. Nucleic AcidsRes.

[13] Xu K, Lu T, Zhou H, Bai L, Xiang Y (2010). The role of MSH5 C85T and MLH3C2531T polymorphisms in the risk of male infertility with azoospermia or severe oligozoospermia. Clin Chim Acta.

[14] Lipkin SM, Wang V, Jacoby R, Banerjee-Basu S, Baxevanis AD, Lynch HT (2000). MLH3: a DNA mismatch repair gene associated with mammalian microsatellite instability. Nat Genet.

[15] Taylor NP, Powell MA, Gibb RK, Rader JS, Huettner PC, Thibodeau SN (2006). MLH3 mutation in endometrial cancer. Cancer Res.

[16] Ng PC, Henikoff S (2001). Predicting deleterious amino acid substitutions. Genome Res.

[17] Ferrás C, Zhou XL, Sousa M, Lindblom A, Barros A (2007). DNA mismatch repair gene hMLH3 variants in meiotic arrest. Fertil Steril.

[18] Kunkel TA, Erie DA (2005). DNA mismatch repair. Annu Rev Biochem.

[19] Iyer RR, Pluciennik A, Burdett V, Modrich PL (2006). DNA mismatch repair: functions and mechanisms. Chem Rev.

[20] Jiricny J (2006). The multifacetedmismatch-repair system. Nature RevMol Cell Biol.

[21] Whitby MC (2005). Making crossovers during meiosis. Biochem Soc Trans.

